# The Impact of Smoking Bans on Clozapine Metabolism and Psychiatric Stability: A Case Report

**DOI:** 10.1192/bjo.2025.10740

**Published:** 2025-06-20

**Authors:** Praveen Kumar, Caio Bezzerraculas, Ananya Santosh, Nikki Thomson

**Affiliations:** 1 City Hospital, Aberdeen, United Kingdom; 2 New Craig’s Psychiatric Hospital, Inverness, United Kingdom

## Abstract

**Aims:** A patient with a diagnosis of schizo-affective disorder, characterized by a history of harmful substance use, was undergoing treatment in the Intensive Psychiatric Care Unit (IPCU). His medical journey, punctuated by numerous admissions since childhood due to various mental health crises, presented considerable treatment challenges. Key components of his therapeutic regimen included clozapine and sodium valproate. His treatment was complicated by a pattern of medication non-compliance, substance misuse, and recurrent hospital admissions. A recent transfer to an unrestricted smoking ward resulted in significant changes in his smoking habits, which notably impacted both his mental health and clozapine levels.

**Methods:** In the IPCU, a patient treated with clozapine for schizo-affective disorder faced notable challenges after the implementation of the Smoke-Free Perimeter Law. Initially permitted a regulated number of cigarettes each day, this allowance was curtailed due to the law’s enforcement requiring staff accompaniment for smoking breaks. The restriction led to reduced cigarette access. Following his transfer to an unrestricted smoking ward, the patient’s cigarette intake increased to 20–30 per day, reverting to his usual habit. This change precipitated a drastic reduction in clozapine levels from 0.46 mg/l to 0.28 mg/l, leading to heightened confusion, delusional thinking, and disorganized speech. Despite no change in medication, it was resolved to move him back to the IPCU under a restricted smoking regimen. As his smoking stabilized at 10–12 cigarettes daily, the clozapine levels fluctuated before eventually normalizing, correlating with a stabilization of his psychiatric symptoms.

**Results:** This case underscores the significant influence of lifestyle habits, especially smoking, on the efficacy of psychiatric medications in mental health care. The increase in cigarette use in the unrestricted ward led to a marked decrease in his clozapine levels, highlighting the interaction between smoking and medication metabolism. The situation points to the crucial role of healthcare providers in closely monitoring and adjusting treatment plans in response to lifestyle changes, ensuring patient well-being alongside adherence to public health policies.

**Conclusion:** This case illustrates the complex challenges posed by the Smoke-Free Perimeter Law in psychiatric care, particularly in terms of personalized care and medication management. The significant changes in the patient’s mental health and clozapine levels following increased smoking in an unrestricted environment underscores the need for careful planning in future transitions. Especially attempts to move the patient to a home or less restricted environment where smoking is more accessible, should be approached cautiously.

